# A sialic acid-targeted near-infrared theranostic for signal activation based intraoperative tumor ablation†
†Electronic supplementary information (ESI) available: All experimental procedures; synthesis and characterization of SA-pNIR and Glu-pNIR; time course studies on cellular uptake of SA-pNIR; metabolic incorporation of SA-pNIR into cellular proteins; and whole body images of mice with overdosed SA-pNIR and Glu-pNIR. See DOI: 10.1039/c4sc02248c



**DOI:** 10.1039/c4sc02248c

**Published:** 2014-09-25

**Authors:** Xuanjun Wu, Mingzhu Yu, Bijuan Lin, Hongjie Xing, Jiahuai Han, Shoufa Han

**Affiliations:** a Department of Chemical Biology , College of Chemistry and Chemical Engineering , the Key Laboratory for Chemical Biology of Fujian Province , The MOE Key Laboratory of Spectrochemical Analysis & Instrumentation , and Innovation Center for Cell Biology , Xiamen University , Xiamen , 361005 , China . Email: shoufa@xmu.edu.cn ; Tel: +86-0592-2181728; b State Key Laboratory of Cellular Stress Biology , Innovation Center for Cell Biology , School of Life Sciences , Xiamen University , Xiamen , 361005 , China

## Abstract

Agents enabling tumor staging are valuable for cancer surgery. Herein, a targetable sialic acid-armed near-infrared profluorophore (SA-pNIR) is reported for fluorescence guided tumor detection. SA-pNIR consists of a sialic acid entity effective for *in vivo* tumor targeting and a profluorophore which undergoes lysosomal acidity-triggered fluorogenic isomerization. SA-pNIR displays a number of advantageous biomedical properties in mice, *e.g.* high tumor-to-normal tissue signal contrast, long-term retention in tumors and low systemic toxicity. In addition, SA-pNIR effectively converts NIR light into cytotoxic heat in cells, suggesting tumor-activatable photothermal therapy. With high performance tumor illumination and lysosome-activatable photothermal properties, SA-pNIR is a promising agent for detection and photothermal ablation of surgically exposed tumors.

## Abstract 2

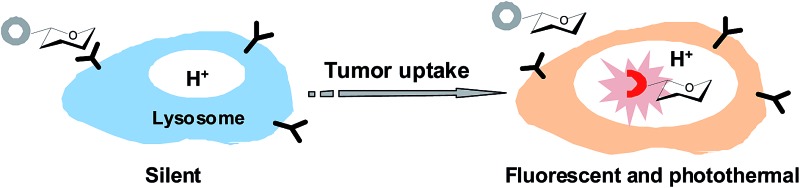
A sialic acid-targeted near-infrared profluorophore with pH-responsive fluorescence and photothermal properties was developed for fluorescence-guided staging and photothermal therapy of viable tumors exposed during surgery.

## Introduction

With the increasing morbidity and mortality imposed by cancers, approaches that could improve the outcome of existing treatment modalities are of significance.^1^ Surgical resection is a mainstay for solid tumor treatment, whereupon residual tumors due to incomplete resection often result in tumor relapse. The utility of fluorescence guided surgery is demonstrated by recent clinical treatment of ovarian cancer with the aid of fluorescein-conjugated folate.^2^ Recently, optical agents that could direct surgeons to tumor foci evasive to visual inspection have been intensively explored.^2,3^

High tumor-to-background signal contrast is paramount for tumor imaging. As such, optical probes that could be activated to a “signal-on” state within tumors while remaining silent in off-target settings are critical to achieve low background signals.^3,4^ Rhodamine derivatives with intramolecular spirorings are poised for proton-triggered fluorogenic opening of the intramolecular rings within acidic lysosomes, enabling high performance tumor imaging in mice models.^5^ Relative to rhodamines, near-infrared (NIR) dyes are advantageous for *in vivo* imaging as biological tissues display the least optical absorption and autofluorescence in the NIR window (650–900 nm).^6^ Nanomaterials that could convert NIR irradiation into cytotoxic heat are being actively explored for photothermal cancer therapy.^7^ Given concerns regarding the biosafety of nanomaterials, small molecular NIR dyes could be biocompatible. For instance, indocyanine green (ICG) has been approved for clinical applications.^8^ Analogous to acid-responsive rhodamines, probes displaying lysosomal acidity-activatable NIR fluorescence and photothermal properties have been largely unexplored for intraoperative tumor therapy.

To achieve the high tumor-to-background signal contrast required for *in vivo* tumor imaging, dyes are often deliberately armed with tumor-homing entities, such as monoclonal antibodies, folates, aptamers, *etc.* ^9^ Sialic acids (SA) are anionic monosaccharides commonly located at termini of cell surface glycans,^10^ and hypersialylation of cell surface constituents has been identified in a broad spectrum of cancers,^11^ suggesting enhanced metabolic demand for SA by these tumors cells. Fluorescein isothiocyanate-labelled sialic acid (SA-FITC) was recently employed for high performance tumor detection in mice, showing effective uptake of SA monosaccharide by metabolically active tumor cells.^12^ Albeit selectively accumulated in tumors, SA-FITC suffers from “always-on” green fluorescence which has limited tissue penetration, and quick *in vivo* clearance which might lead to fast attenuation of tumor-associated signals during surgery.^12^ Herein, we report a sialylated pH-activatable NIR profluorophore (SA-pNIR) for targeted tumor imaging and photothermal therapy in mice with dramatically improved pharmacokinetics critical for clinical translation. SA-pNIR consists of a sialic acid entity for effective *in vivo* tumor uptake and an activatable NIR profluorophore which becomes photothermal and fluorescent within acidic lysosomes.

## Results and discussion

### Acidic pH mediated fluorescence activation of SA-pNIR

**Fig. 1:**
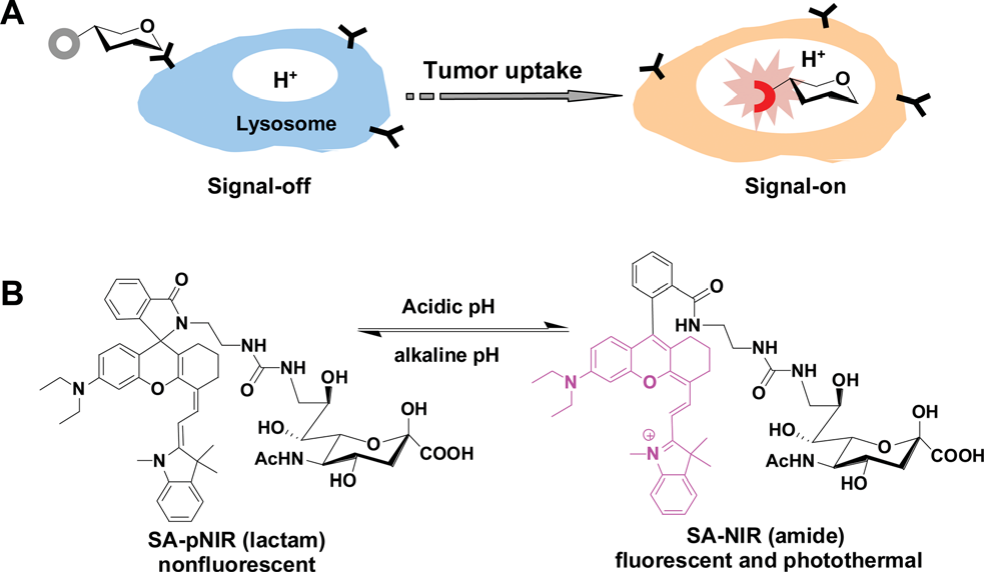
Schematic of tumor illumination with a targetable sialic acid-conjugated NIR profluorophore (SA-pNIR) activatable to lysosomal acidity (A); nonfluorescent SA-pNIR undergoes acidic pH-mediated fluorogenic opening of the intramolecular lactam to give fluorescent and photothermal SA-NIR (B).

**Fig. 2:**
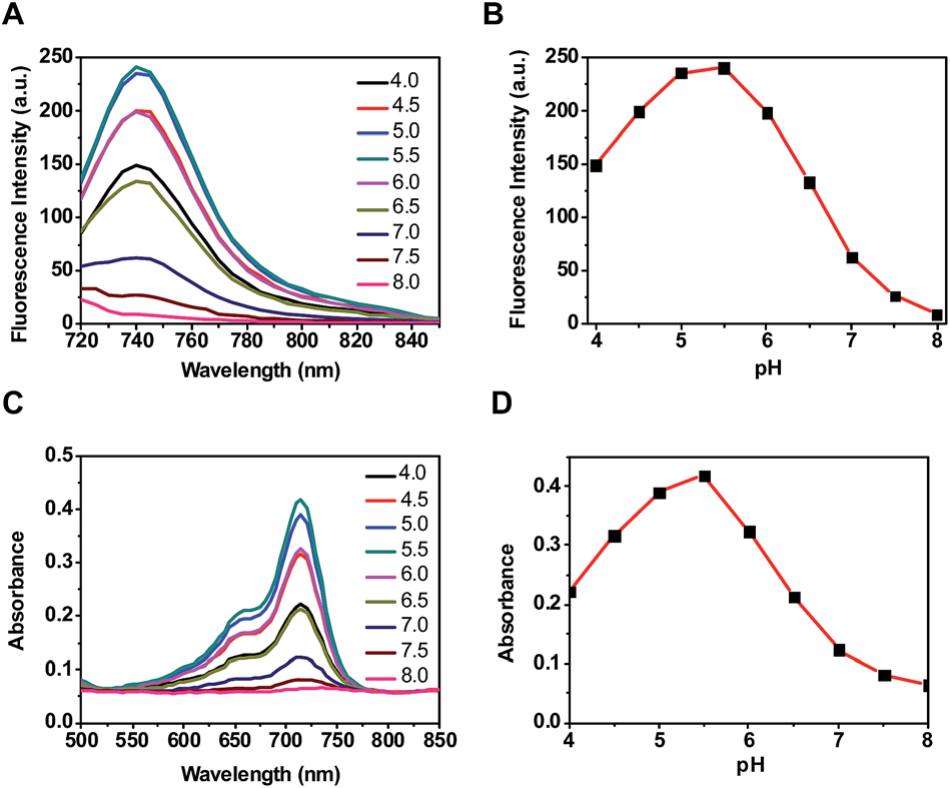
pH responsiveness of SA-pNIR. SA-pNIR was spiked into sodium phosphate buffer (100 mM, pH 4.0–8.0) containing 10% acetonitrile (v/v) to give a final concentration of 10 μM. Fluorescence emission of the solutions was recorded using *λ*_ex_@715 nm (A) and the fluorescence intensities at 740 nm were plotted against buffer pH (B). UV-vis-NIR absorption spectra of the solutions were collected over the buffer pH range (C) and the absorbance at 715 nm was plotted as a function of buffer pH (D).

Optical probes with turn-on fluorescence inside tumors while being silent in off-target settings are beneficial for low-background tumor imaging.^3,4^ We recently reported the use of rhodamines with intramolecular spirorings for *in vivo* tumor detection *via* lysosomal acidity-triggered fluorogenic opening of the rings to give rhodamines.^5^ Relative to red-emissive rhodamines, NIR dyes are advantageous for bioimaging due to the enhanced tissue penetration of NIR fluorescence and the minimal light absorption and autofluorescence of biological tissues in the NIR region.^6^ Hence, we set out to develop a NIR probe, akin to rhodamine–lactams, for tumor imaging *via* lysosomal acidity-mediated fluorescence activation within tumors (Fig. 1B).

((*E*)-2-(2-(9-(2-Carboxyphenyl)-6-(diethyl-amino)-2,3-dihydro-1*H*-xanthen-4-yl)vinyl)-1,3,3-trimethyl-3*H*-indol-1-ium perchlorate), a NIR dye reported by Lin *et al.*,^13^ was first amidated with ethylenediamine and then conjugated with 9-amino-9-deoxy-5-*N*-acetylneuraminic acid to afford the desired SA-pNIR in 32% overall yield (ESI†). To probe its pH responsiveness, SA-pNIR was spiked into buffers of pH 4.0–8.0 and the fluorescence emission and UV-vis-NIR absorption of the solutions were recorded over the buffer pH range. As shown in Fig. 2, SA-pNIR exhibits NIR absorption and fluorescence emission in acidic media, proving the proton-mediated isomerization of SA-pNIR into fluorescent SA-NIR as shown in Fig. 1B. pH titration shows that SA-pNIR is weakly fluorescent at cytosolic pH (pH 7.2) and yet exhibits enhanced fluorescence at pH 4.5–6.0, which ideally matches the lysosomal pH window (pH 4–6). These results indicate the applicability of SA-pNIR to illuminating acidic lysosomes in living cells.

### Illumination of acidic lysosomes in cells by SA-pNIR

**Fig. 3:**
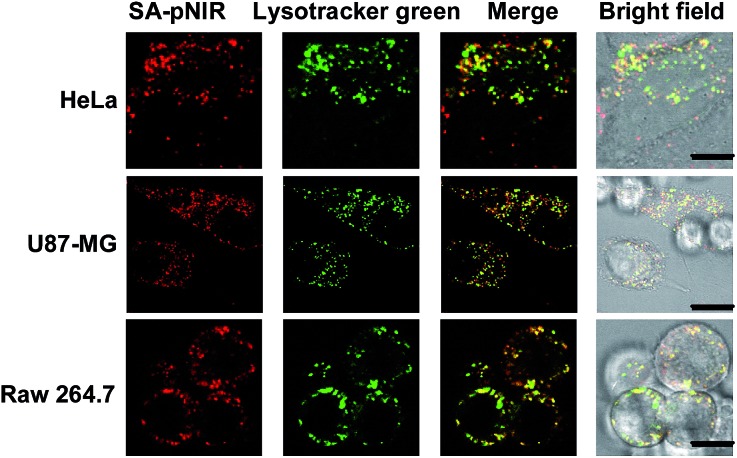
Lysosome illumination with SA-pNIR. HeLa, U87-MG and Raw 264.7 cells were cultured for 1 h with SA-pNIR (100 μM) in DMEM and then stained with Lysotracker green (1 μM) for 20 min. The cells were probed by confocal fluorescence microscopy. Merging of the SA-NIR signal (shown in red) and that of Lysotracker green (shown in green) revealed colocalization, as indicated by the yellow areas. Bars, 10 μm.

Lysosomes are the major intracellular acidic compartments and the number and acidity of lysosomes could be significantly boosted in cancer cells.^14^ To determine the performance of intracellular signal activation, HeLa cells, U87-MG cells and Raw 264.7 cells were cultured in Dulbecco's Modified Eagle's Medium (DMEM) supplemented with SA-pNIR and then stained with LysoTracker Green DND-26, referred to herein as Lysotracker green. Confocal microscopy images show that NIR fluorescence is clearly observed within all the three cell lines tested and colocalizes with Lysotracker green specific for acidic lysosomes (Fig. 3). The colocalization confirms that SA-pNIR could be taken up by these cells and then activated to fluorescent SA-NIR within lysosomes. To probe cellular uptake kinetics, HeLa, U87-MG and Raw 264.7 cells were loaded with SA-pNIR and then stained with DiI specific for plasma membranes. The intracellular NIR signals were determined after incubation for 1 h, 4 h and 24 h. It was revealed that the intracellular NIR fluorescence intensified upon prolonged incubation (Fig. S1, ESI†), suggesting continuous uptake of SA-pNIR from the surrounding medium by these cell lines.

**Fig. 4:**
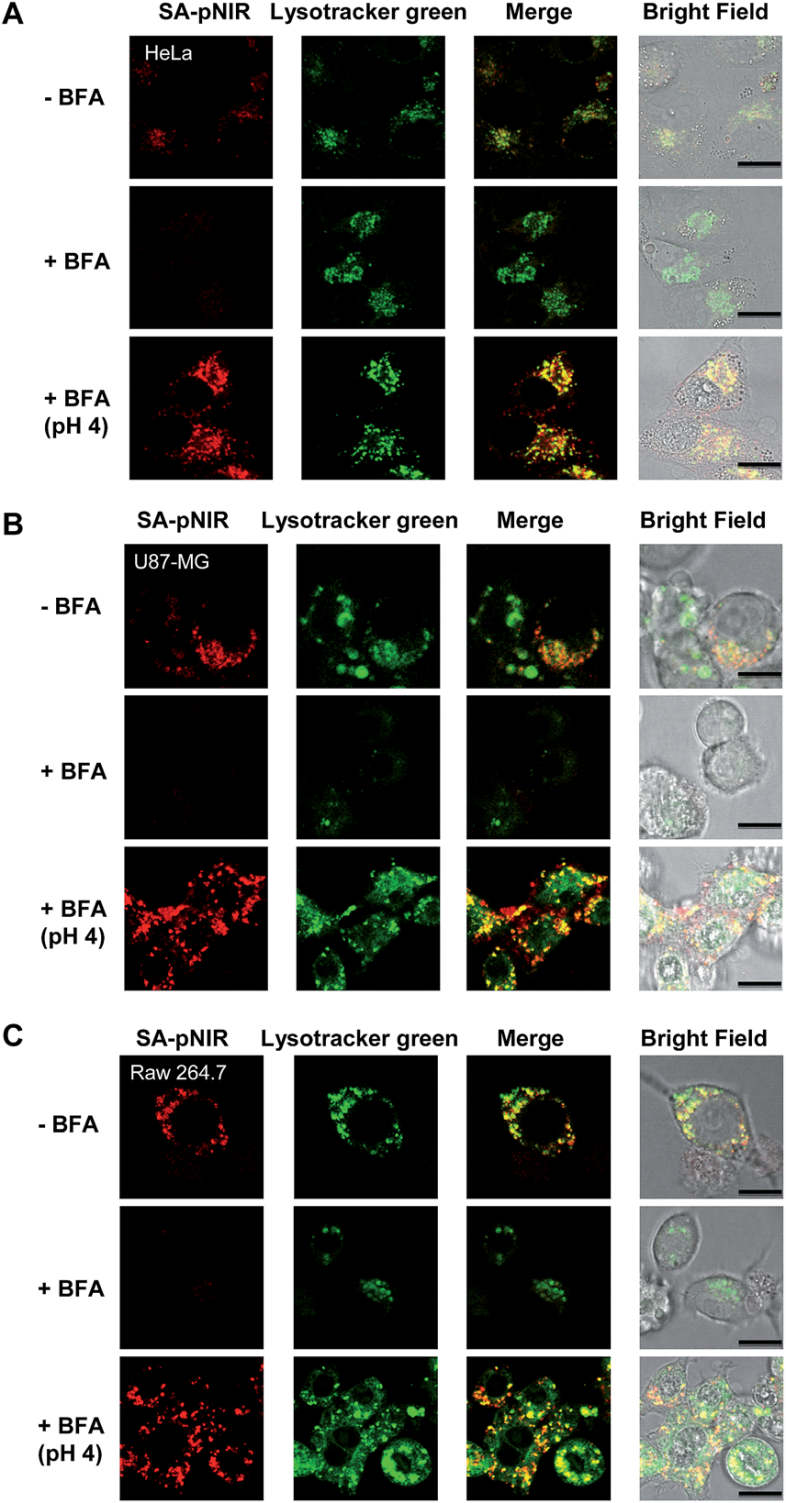
Acidic pH-mediated fluorescence-on of SA-pNIR within cells. HeLa (A), U87-MG (B) and Raw 264.7 (C) cells with or without BFA (50 nM) pretreatment were cultured with SA-pNIR (100 μM) in DMEM for 1 h and then stained with Lysotracker green (1 μM) in DMEM for 20 min. For the control experiment, cells loaded with BFA and SA-pNIR were resuspended in sodium phosphate buffer (pH 4, 100 mM) for 10 min. The cells were visualized by confocal fluorescence microscopy. The intracellular NIR signals were merged with Lysotracker green and the colocalization is shown by the yellow areas. Bars, 10 μm.

To assess the dependence of intracellular NIR fluorescence on lysosomal acidity, we measured SA-pNIR signals in cells treated with Bafilomycin A1 (BFA), which is a potent inhibitor of V-ATPase and effectively alkalinizes lysosomes in BFA-treated cells.^15^ As shown in Fig. 4, the intracellular signals of SA-pNIR largely vanished in HeLa, U87-MG and Raw 264.7 cells pretreated with BFA compared to the cells in the absence of BFA. The BFA- and SA-pNIR treated cells that displayed markedly decreased NIR signals within the cells were further incubated in a phosphate buffer of pH 4.0. Fig. 4 revealed the recovery of intense intracellular NIR signals in the aforementioned HeLa, U87-MG and Raw 264.7 cells in acidic buffer, excluding the loss of intracellular SA-pNIR in BFA-treated cells and further confirming the lysosomal pH dependent “turn-on” fluorescence of SA-pNIR in cells. Solid tumors are hallmarked by acidic microenvironments due to metabolically accumulated lactic acid.^16^ The acidic microenvironment has been widely targeted for tumor therapy and imaging. The recovered NIR fluorescence of BFA-treated cells in acidic buffer strongly indicates that the acidic tumor microenvironments and tumor lysosomes might exert synergistic effects on signal activation of SA-pNIR endocytosed into tumor cells *in vivo*.

### Illumination of subcutaneous tumors in mice with SA-pNIR

**Fig. 5:**
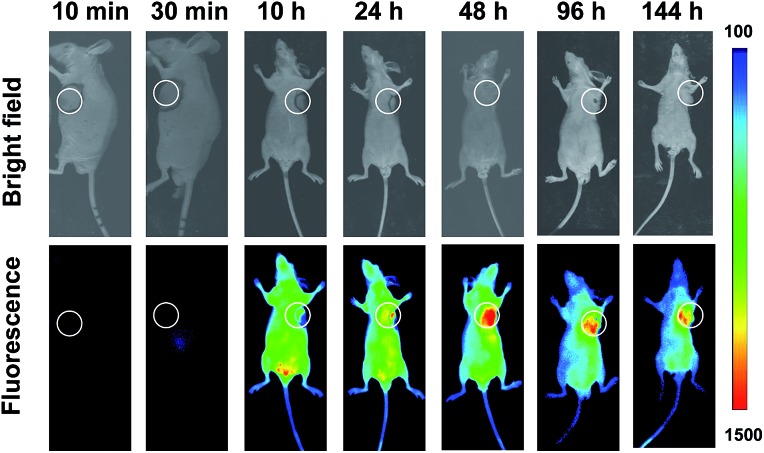
Time-lapse illumination of subcutaneous tumors with SA-pNIR. Nude mice bearing subcutaneous tumors were intravenously injected with SA-pNIR (40 mg kg^–1^) *via* tail vein and then imaged for *in vivo* NIR fluorescence emission at the indicated time points.

Shown to fluoresce in lysosomes, SA-pNIR was further evaluated for its efficacy and selectivity in illuminating subcutaneous tumors in mice. Nude or ICR mice were inoculated subcutaneously with H22 hepatocellular carcinoma cells and then maintained for 5–10 days to allow the development of tumor xenografts. SA-pNIR were intravenously administered into the tumor-bearing nude mice *via* tail vein. The mice were imaged for whole body NIR fluorescence over the course of 144 h after injection. No NIR signal was observed in mice 30 min after administration (Fig. 5), demonstrating that SA-pNIR remained silent during circulation in the blood stream (pH 7.4). Intense NIR fluorescence was identified in subcutaneous tumors at 48 h postinjection and the signal contrast between tumor and normal tissues remained high up to 144 h postinjection (Fig. 5). The tumor-associated fluorescence validates that SA-pNIR is effectively accumulated in tumors, where it is activated to the “signal-on” state.

**Fig. 6:**
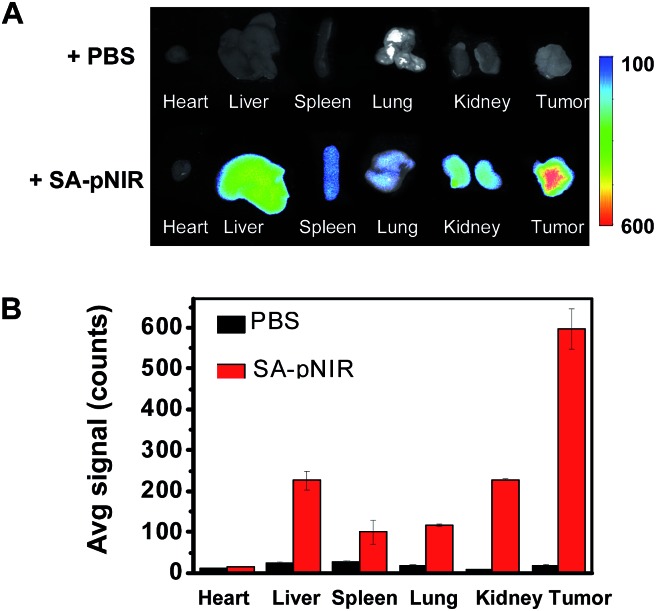
Biodistribution of SA-pNIR in tumor-bearing mice. ICR mice bearing subcutaneous tumors were intravenously injected with SA-pNIR (40 mg kg^–1^) or PBS (100 μl) and then sacrificed 48 h postinjection. The tumor and representative organs were dissected and then imaged for *ex vivo* fluorescence emission (A). The bar graph shows the tissue-specific NIR fluorescence intensity (B).

To further determine the biodistribution of SA-pNIR in subcutaneous tumors and healthy tissues, the tumor and representative organs were dissected from tumor-bearing ICR mice pretreated with SA-pNIR for 48 h and then probed by *ex vivo* fluorescence analysis. Consistently, intense signals were observed in the tumor whereas moderate to low levels of NIR fluorescence were present in the kidney, heart, spleen, lung and liver (Fig. 6B), validating effective tumor uptake and activation of SA-pNIR within tumors. The liver- and kidney-associated NIR fluorescence suggests renal and hepatic clearance of injected SA-pNIR, which is beneficial for clinical translation. Collectively, these data confirm preferential *in vivo* tumoral uptake of SA-pNIR and ensuing fluorescence activation of SA-pNIR, which correlate well with the aforementioned whole body imaging studies (Fig. 5).

In previous tumor imaging studies, the fluorescence of SA-FITC within tumors reached maxima at 20 min postinjection and then quickly decreased by 80% at 1 h postinjection.^12^ The long-term retention of SA-pNIR within tumors together with the preferential tumor accumulation of SA-pNIR and the high tumor-to-healthy tissue signal ratios suggest the potential utility of SA-pNIR for low background intraoperative tumor detection.

**Fig. 7:**
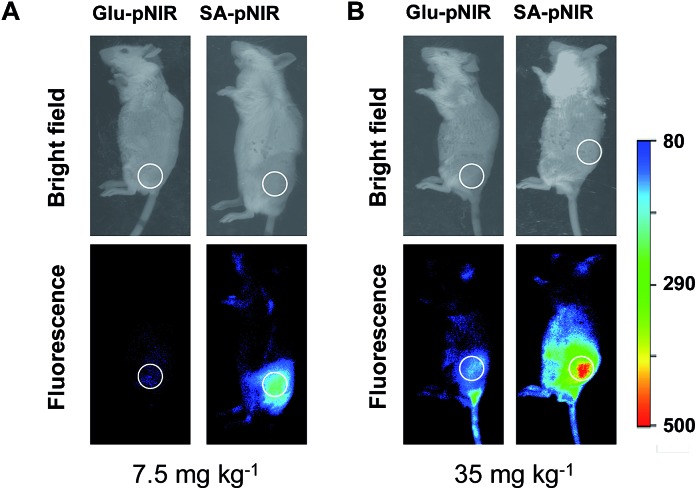
Sialic acid-mediated tumoral accumulation of SA-pNIR in mice. Tumor-bearing ICR mice were intravenously injected with SA-pNIR or Glu-pNIR with doses of 7.5 mg kg^–1^ (A) or 35 mg kg^–1^ (B) and then imaged 48 h postinjection.

To probe the impact of the sialic acid domain of SA-pNIR on *in vivo* tumor targeting, d-glucosamine conjugated with the NIR profluorophore (Glu-pNIR) was prepared and then administered into tumor-bearing ICR mice *via* tail vein (ESI†). In contrast with mice treated with SA-pNIR, whole body imaging revealed no significant NIR signal in subcutaneous tumors from mice treated with Glu-pNIR at 48 and 144 h postinjection (Fig. 7 and S3, ESI†), demonstrating the critical role of sialic acid for tumor targeting. Historically, monoclonal antibodies, folates, peptides and aptamers have often been used to direct dyes to target tumors.^9^ The demonstrated high performance tumor illumination with SA-pNIR shows that sialic acid with a C-9 conjugated theranostic entity is an attractive warhead for targetable cancer imaging.

Hypersialylation of cell surface glycoconjugates is a hallmark of a broad spectrum of cancers^11^ and often correlates with their metastatic potentials.^17^ The tumor-associated over-sialylation suggests enhanced metabolic demand for SA by tumors. Historically, metabolic engineering of cell surface sialosides has been achieved with exogenous *N*-acyl mannosamines, the metabolic precursor of SA.^18^ However, this approach is limited by low cell type- or tissue-specificity, as demonstrated by broad expression of metabolically synthesized SA in different tissues from supplemented *N*-acyl mannosamines in animals.^19^ In contrast, SA-FITC displays a high tendency to recognize liver tumors in mice.^12^ Albeit preferentially and quickly accumulating in tumors in mice, SA-FITC undergoes quick *in vivo* clearance, leading to significant fluorescence-off within tumors. The distinct biomedical properties of SA-pNIR as compared to SA-FITC, *e.g.* long-term tumoral retention, clearly demonstrate the beneficial effects of the pNIR moiety on *in vivo* tumor illumination. These observations reveal that the *in vivo* pharmacokinetics of sialic acid-conjugated theranostics could be effectively modulated with substituents of appropriate hydrophobicity (*i.e.* pNIR *vs.* FITC) at the C-9 position of sialic acid.

### Cytotoxicity of SA-pNIR

**Fig. 8:**
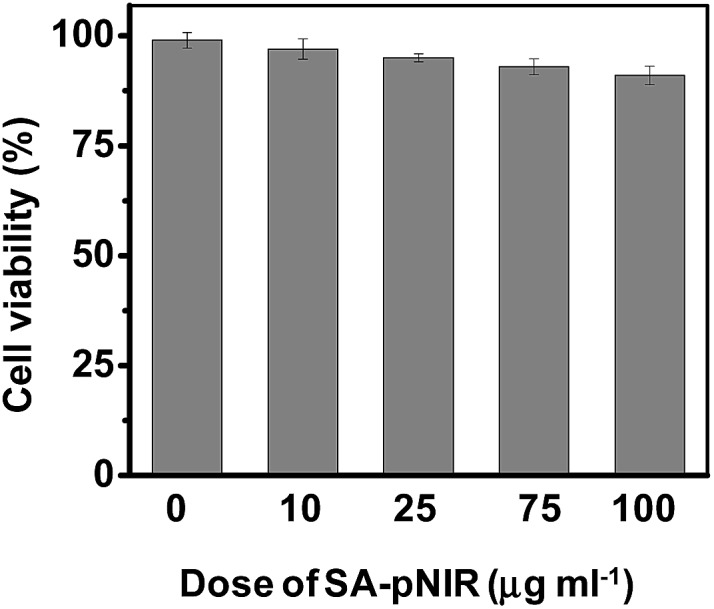
Cytotoxicity of SA-pNIR. HeLa cells were cultured in DMEM containing various levels of SA-pNIR (0–100 μg mL^–1^) for 24 h. Cell number and cell viability were determined by trypan blue exclusion assay.

Low toxicity is a prerequisite for imaging agents aimed at *in vivo* administration. We first examined the effects of SA-pNIR on the survival of HeLa cells by trypan blue exclusion test. No obvious detrimental effects on cell viability were observed on cells treated with SA-pNIR for 24 h at doses up to 100 μg mL^–1^ (Fig. 8), suggesting low cytotoxicity of SA-pNIR. To probe the systemic toxicity, SA-pNIR was injected into healthy mice by tail vein at doses of 150 mg kg^–1^. The mice were regularly monitored for adverse effects following injection. No signs of abnormality, including death, pain or fatigue, were observed in the probe-treated mice up to 10 days after injection. *Ex vivo* analysis revealed low levels of NIR fluorescence in the organs excised from the mice (ESI,† Fig. S5 and S6), suggesting that SA-pNIR could be effectively cleared from the body. Taken together, these results suggest that SA-pNIR is of low biotoxicity.

### Acidic pH dependent photothermal effects of SA-pNIR

**Fig. 9:**
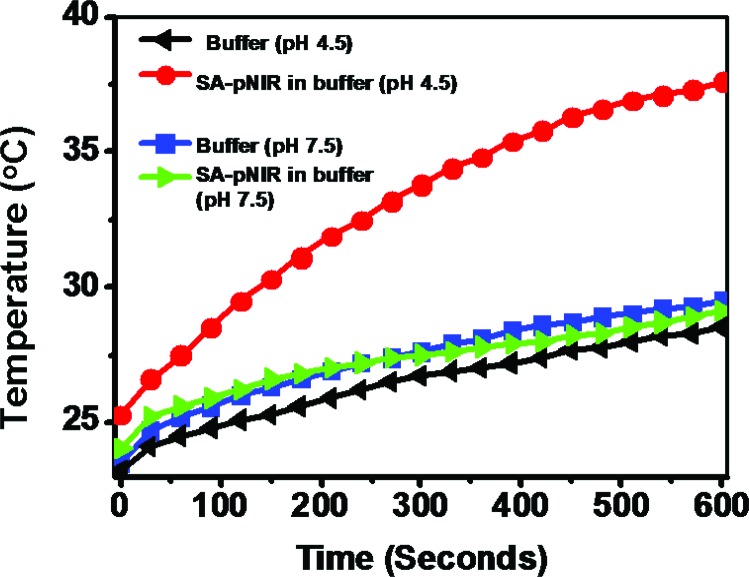
Acidic pH-dependent photothermal properties of SA-pNIR. The temperature of sodium phosphate buffer (100 mM, pH 4.5 or 7.5) containing SA-pNIR (0 or 0.1 mg mL^–1^) was recorded over the time of irradiation with an NIR laser (660 nm, 0.5 W cm^–2^).

Reagents that could convert optical energy into cytotoxic heat are attractive tools for light-mediated photothermal tumor therapy.^7^ As such, intense investigations have been devoted to the development of various NIR-absorbing nanomaterials.^7^ Given long-standing concerns regarding the *in vivo* biosafety of nanoscaled materials, small molecule theranostics are suitable for *in vivo* studies, as demonstrated by the approval of indocyanine green dye (ICG) for clinical applications. Inspired by the emerging use of NIR dyes in photothermal therapy,^20^ we proceeded to examine the capability of SA-pNIR as a pH-responsive photothermal agent. SA-pNIR was spiked into buffers of pH 7.5 and 4.5. The solutions were exposed to 660 nm laser illumination at a power density of 0.5 W cm^–2^ and the temperature of the solutions was monitored over the irradiation time. Fig. 9 clearly shows temperature elevation dependent on the acidic pH, demonstrating that SA-pNIR effectively converts NIR irradiation into heat in acidic media.

### SA-pNIR mediated photothermal killing of cells

**Fig. 10:**
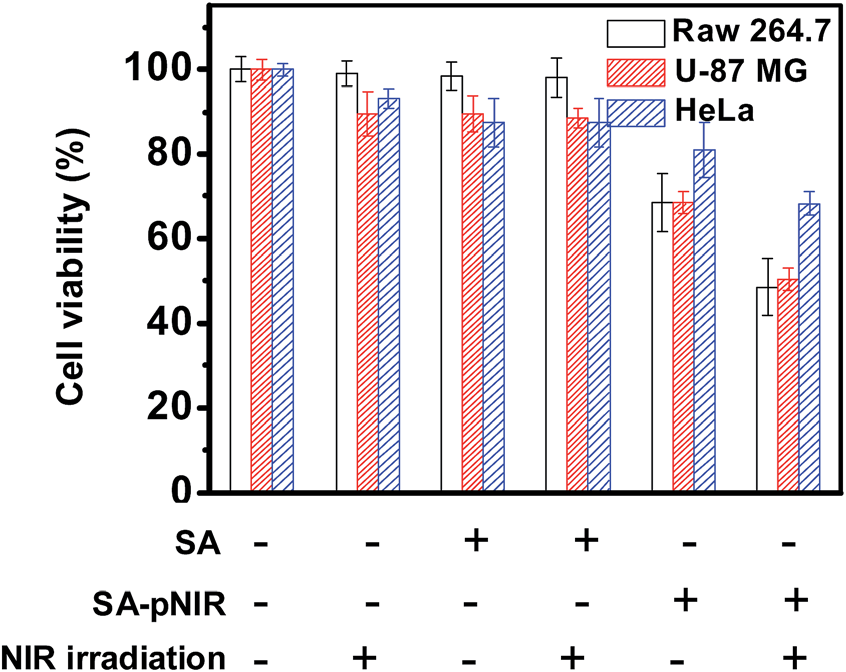
Photothermal effects of SA-pNIR on live cells. HeLa, U87-MG and Raw 264.7 cells were cultured for 24 h with SA-pNIR (0 or 100 μg mL^–1^) or SA (0 or 100 μg mL^–1^) in DMEM and then either irradiated with an NIR laser (660 nm, 0.5 W cm^–2^), or not subjected to irradiation, in fresh DMEM for 10 min, and then further cultured for 24 h. Cell viability was determined by MTT assay.

SA-pNIR was then evaluated for its photothermal effects on host cells. HeLa, U87-MG and Raw 264.7 cells pre-loaded with SA-pNIR or SA were either irradiated with an NIR laser, or not subjected to irradiation. The viability of these cell populations was assayed using 3-(4,5-dimethylthiazol-2-yl)-2,5-diphenyltetrazolium (MTT). Cells treated with SA-pNIR and light illumination display further decreased viability relative to that of cells treated with NIR laser irradiation or SA-pNIR alone (Fig. 10), demonstrating the synergistic effects of SA-pNIR and light irradiation for detrimental effects on host cells. SA-pNIR has a maximal absorption at 715 nm (Fig. 1). The molecular extinction coefficient at 715 nm is 2-fold higher than that at 660 nm. Given the suboptimal wavelength used for photothermal evaluation of SA-pNIR (660 nm, Fig. 9 and 10), it can be anticipated that the efficacy of SA-pNIR mediated photothermal killing of tumor cells could be further increased with an appropriate instrumental laser (715 nm).

Complete manual cytoreduction of small-sized or embedded tumor foci is often challenging during surgery. Photothermal tumor ablation during surgery is applicable due to surgical exposure of cancerous tissues that are otherwise inaccessible to exogenous laser irradiation. Theranostics allowing intraoperative tumor staging and simultaneous photothermal tumor therapy are of clinical significance to complement surgical dissection. The lysosomal acidity-triggered photothermal effect of SA-pNIR on targeted cells supports the potential utility of SA-pNIR as a theranostic probe for dual imaging and photothermal killing of tumor foci in intraoperative settings.

## Conclusions

SA-pNIR, a sialylated lysosome-activatable NIR dye, has been developed for intraoperative tumor therapy. The sialic acid entity enables effective tumor targeting in mice and the NIR profluorophore undergoes lysosomal pH-triggered isomerization to give an NIR signal. In contrast with SA-FITC which is compromised by “always-on” green fluorescence and quick *in vivo* clearance, SA-pNIR displays signal activation in viable tumor cells, high tumor-to-normal tissue signal contrasts, and long-term retention in tumors, rendering optical imaging over an adequate duration which is critical for practical surgical intervention. In addition, SA-pNIR effectively converts NIR irradiation into heat in acidic lysosomes and leads to obvious cell death upon NIR irradiation, suggesting its utility for photothermal ablation of surgically exposed tumor foci that are otherwise inaccessible to exogenous light. With superior *in vivo* pharmacokinetics, high performance tumor illumination, and acid-responsive photothermal properties, SA-pNIR is a promising small molecular theranostic for fluorescence guided tumor detection and possibly photothermal tumor therapy in intraoperative settings.

## References

[cit1] Nguyen Q. T., Tsien R. Y. (2013). Nat. Rev. Cancer.

[cit2] van Dam G. M., Themelis G., Crane L. M., Harlaar N. J., Pleijhuis R. G., Kelder W., Sarantopoulos A., de Jong J. S., Arts H. J., van der Zee A. G., Bart J., Low P. S., Ntziachristos V. (2011). Nat. Med..

[cit3] Lee H., Akers W., Bhushan K., Bloch S., Sudlow G., Tang R., Achilefu S. (2011). Bioconjugate Chem..

[cit4] Kobayashi H., Choyke P. L. (2011). Acc. Chem. Res..

[cit5] Wu X., Tian Y., Yu M., Han J., Han S. (2014). Biomater. Sci..

[cit6] Weissleder R., Ntziachristos V. (2003). Nat. Med..

[cit7] Dickerson E. B., Dreaden E. C., Huang X., El-Sayed I. H., Chu H., Pushpanketh S., McDonald J. F., El-Sayed M. A. (2008). Cancer Lett..

[cit8] Malicka J., Gryczynski I., Geddes C. D., Lakowicz J. R. (2003). J. Biomed. Opt..

[cit9] Low P. S., Henne W. A., Doorneweerd D. D. (2008). Acc. Chem. Res..

[cit10] Angata T., Varki A. (2002). Chem. Rev..

[cit11] Hakomori S. (1996). Cancer Res..

[cit12] Wu X., Tian Y., Yu M., Lin B., Han J., Han S. (2014). Biomater. Sci..

[cit13] Yuan L., Lin W., Yang Y., Chen H. (2012). J. Am. Chem. Soc..

[cit14] Kroemer G., Jaattela M. (2005). Nat. Rev. Cancer.

[cit15] Yoshimori T., Yamamoto A., Moriyama Y., Futai M., Tashiro Y. (1991). J. Biol. Chem..

[cit16] Gatenby R. A., Gillies R. J. (2004). Nat. Rev. Cancer.

[cit17] Bernacki R. J., Kim U. (1977). Science.

[cit18] Luchansky S. J., Goon S., Bertozzi C. R. (2004). ChemBioChem.

[cit19] Kayser H., Zeitler R., Kannicht C., Grunow D., Nuck R., Reutter W. (1992). J. Biol. Chem..

[cit20] Zheng M., Yue C., Ma Y., Gong P., Zhao P., Zheng C., Sheng Z., Zhang P., Wang Z., Cai L. (2013). ACS Nano.

