# Supplementary material for: A sialic acid-targeted near-infrared theranostic for signal activation based intraoperative tumor ablation
Source: Chem Sci. 2014 Sep 25;6(1):798–803. doi: 10.1039/c4sc02248c (PMC5494541; doi:10.1039/c4sc02248c)
Supplement: Supplementary file 1 [file SC-006-C4SC02248C-s001.pdf]

Electronic Supplementary Material (ESI) for Chemical Science.
This journal is © The Royal Society of Chemistry 2014

Supporting information

A sialic acid-targeted near-infrared theranostic for signal activation based intraoperative tumor ablation

Xuanjun Wu,[a] Mingzhu Yu,[a] Bijuan Lin,[a] Hongjie Xing,[a] Jiahuai Han,[b] and Shoufa Han[a,*]

[a]*Department of Chemical Biology, College of Chemistry and Chemical Engineering, the Key Laboratory for Chemical Biology of Fujian Province, The MOE Key Laboratory of Spectrochemical Analysis & Instrumentation, and Innovation Center for Cell Biology, Xiamen University;* [b]*State key Laboratory of Cellular Stress Biology, Innovation Center for Cell Biology, School of Life Sciences, Xiamen University, Xiamen, 361005, China; Tel: 86-0592-2181728; E-mail:* shoufa@xmu.edu.cn

Experimental

Material and methods

LysoTracker Green DND-26 was purchased from Invitrogen. 1,1'-Dioctadecyl-3,3,3',3'-tetramethylindocarbocyanine perchlorate (DiI) and 3-(4,5-dimethylthiazol-2-yl)-2,5-diphenyltetrazolium bromide (MTT) were obtained from Beyotime. Dulbecco's Modified Eagle Medium (DMEM) and GIBCO fetal bovine serum (FBS) were purchased from Sigma-Aldrich. Changsha Red was synthesized according to a published procedure.[12] 9-Amino-9-deoxy-5-N-acetylneuraminic acid was prepared following a published procedure.[20] FBS was heat-inactivated at 56 °C for 30 min before use. All other chemicals were used as received from Alfa Aesar. The UV-vis-NIR absorbance spectra and fluorescence spectra were collected at a spectrofluorimeter (Spectramax M5, Molecular Device).

U87-MG cells, HeLa cells, Raw 264.7 cells were obtained from American Type Culture Collection and grown at 37 °C under 5% CO_2 in DMEM. H22 hepatocellular carcinoma cells were obtained from peritoneal cavity of tumor-bearing mice and used for inoculation of tumors in mice. Confocal microscopic images were performed on Leica SP5 using the following filters: λex@488 nm and λem@500-530 nm for Lysotracker green; λex@543 nm and λem@555-600 nm for DiI; λex@633 nm and λem@700-790 nm for SA-pNIR. Fluorescence images were merged by Photoshop CS 6.0. Spectra data were processed with origin 8.5 software. Fluorescence imaging of mice and the dissected organs were obtained on Carestream FX PRO *in vivo* imaging system using an excitation filter of 690 nm and an emission filter of 750 nm. Fluorescence quantification data were analyzed with Carestream MI SE software. Both nude and ICR mice were purchased from Xiamen University Laboratory Animal Center. All animal experiments were performed in accordance with the guidelines of Xiamen University's Animal Care and Use Committee.

Synthesis of pNIR (Compound 1)

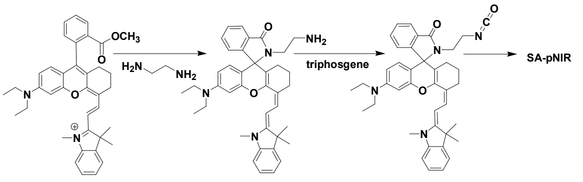

Ester of Changsha Red Compound 1 compound 2

Scheme S1. Synthesis of SA-pNIR.

Changsha Red (10 g) was added to a flask containing methanol (100 ml). To the solution in ice bath was added thionyl chloride (10 ml) dropwise. The resultant solution was heated at 70 ℃ overnight. The solvent was removed by rotary evaporation, and the residue was dissolved in methanol (20 ml). To the solution was added ethylenediamine (20 ml). The reaction solution was heated at 70 ℃ for 1 h and then concentrated by evaporation. The residue was purified by silica gel chromatography using dichloromethane/hexanes/triethylamine (10:10:1) as the eluent to give compound **1** as a pale yellow solid (4 g, 37%). ^{1}H-NMR (400 MHz, DMSO-d_6): δ 7.74 (d, 1H, J = 6.96), 7.53-7.40 (m, 3H), 7.24 (d, 1H, J = 7.04), 7.12 (t, 2H, J = 7.56), 6.78 (q, 2H, J = 5.04), 6.37-6.21 (m, 3H), 5.39 (d, 1H, J = 12.68), 3.31 (q, 4H, J = 6.96), 3.13 (s, 3H), 3.10-3.02 (m, 2H), 2.60 (m, 1H), 2.50-2.43 (m, 1H), 1.63 (d, 6H, J = 3.56), 1.57-1.48 (m, 3H), 1.37-1.18 (m, 3H), 1.09 (t, 6H, J = 7.00); ^{13}C-NMR (100 MHz, DMSO-d_6): 167.72, 157.75, 152.48, 151.65, 148.65, 147.56, 145.41, 138.61, 132.82, 131.88, 128.77, 128.59, 128.14, 123.67, 122.66, 122.00, 119.75, 119.70, 108.81, 106.56, 105.28, 103.90, 97.26, 92.41, 66.21, 55.34, 45.34, 44.10, 43.53, 29.33, 28.54, 28.43, 25.20, 12.19; HRMS ($C_{39}H_{44}N_4O_2$): calculated ($M+H^+$): 601.3537, found: 601.3543.

Synthesis of SA-pNIR

Compound **1** (1 g) dissolved in *N,N*-dimethylformamide (10 ml) was added dropwise to a flask containing triphosgene (500 mg) and dichloromethane (15 ml) at ice bath. To the stirred solution was then added triethylamine (1 ml). The resultant solution was stirred at rt for 10 min. The solvent was removed by rotary evaporation *in vacuo* to afford the crude compound **2**. The slolution of crude compound **2** in DMF (10 ml) containing triethylamine (1 ml) was dropwise added to the solution of 9-Amino-9-deoxy-5-N-acetylneuraminic acid (9-amino-SA, 500 mg) dissolved in methanol (10 ml). The reaction mixture was stirred at rt for 30 min and then concentrated by rotary evaporation. The residue was purified by silica gel chromatography using dichloromethane/ methanol/ triethylamine (10:10:1) as the eluent. The resultant SA-pNIR was further purified by high performance liquid chromatography (HPLC) using H_2O/acetonitrile (V/V: 2:3) to afford SA-pNIR (0.5 g, 32%). ^{1}H-NMR (400 MHz, DMSO-d_6): δ 7.74 (d, 1H, J = 6.52), 7.50 (dd, 1H, J_1 = 6.80, J_2 = 6.52), 7.40 (d, 1H, J = 11.60), 7.27 (d, 1H, J = 6.04), 7.14 (d, 1H, J = 6.04), 6.78 (s, 1 H), 6.34-5.66 (m, 3H), 5.39 (d, 1H, J = 13.20), 5.09 (s, 1H), 4.95 (s, 1H), 4.78 (s, 1H), 3.90-3.67 (m, 3H), 3.68-3.52 (m, 2H), 3.31 (s, 4H), 3.15 (d, 6H, J = 10.00), 2.99-2.81 (m, 2H), 2.49 (s, 3H), 1.98-1.77 (m, 5H), 1.63 (d, 4H, J = 6.80), 1.12-1.07 (m, 2H), 1.03 (t, 12H, J = 8.00); ^{13}C-NMR (100 MHz, DMSO-d_6): 174.65, 171.85, 167.97, 159.28, 157.82, 152.44, 151.70, 148.71, 147.63, 145.40, 138.62, 133.07, 131.51, 128.88, 128.51, 128.16, 123.70, 122.80, 122.04, 119.68, 108.93, 106.60, 104.86, 103.66, 97.27, 96.17, 92.40, 70.85, 69.32, 66.40, 53.46, 49.01, 45.35, 44.09, 41.19, 38.37, 34.45, 29.36, 28.58, 28.46, 25.17, 23.09, 22.13, 12.79; HRMS ($C_{51}H_{62}N_6O_{11}$): calculated ($M-H^+$): 933.4404, found: 933.4302.

Synthesis of Glu-pNIR

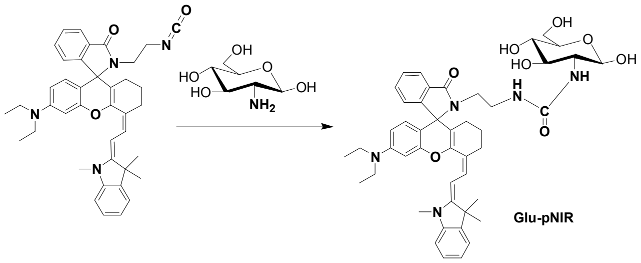

Scheme S2. Synthesis of Glu-pNIR.

DMF (10 ml) containing crude compound **2** (1 g) was added dropwise to was added D-gluocosamine (500 mg) pre-dissolved in methanol (15 ml). Triethylamine (1 ml) were added to the aforementioned solution and the reaction solution was stirred at rt for 30 min and evaporated *in vacuo*. The residue was purified by silica gel chromatography using dichloromethane/ methanol/ triethylamine (10:10:1) as the eluent to afford Glu-pNIR (0.5 g, 36%). ^{1}H-NMR (500 MHz, methanol-d$_4$): δ 7.84 (d, 1H, J = 7.45), 7.62 (t, 1H, J = 7.30), 7.54 (q, 2H, J = 7.45), 7.24-7.15 (m, 3H), 6.84 (t, 1H, J = 7.20), 6.73 (d, 1H, J = 7.75), 6.44-6.32 (m, 3H), 5.45 (d, 1H, J = 12.50), 3.84-3.76 (m, 2H), 3.74-3.70 (m, 2H), 3.65-3.58 (m, 2H), 3.37 (s, 3H), 3.33 (t, 4H, J = 1.55), 3.10-3.06 (m, 4H), 1.71 (d, 6H, J = 1.50), 1.67-1.63 (m, 2H), 1.48 (t, 2H, J = 7.35), 1.20 (t, 6H, J = 7.05); HRMS ($C_{46}H_{55}N_5O_8$): calculated (M+H$^+$): 828.3943, found: 828.3942.

pH titration of SA-pNIR or Glu-pNIR

Aliquots of stock solution of SA-pNIR (10 µl, 1 mM in DMF), Glu-pNIR (10 µl, 1 mM in DMF) were respectively added to sodium phosphate buffers (100 mM, 1 ml) of various pH for 15 min containing 10% acetonitrile (V/V). The fluorescence emission of the solutions was recorded as a function of buffer pH using *λex*@715 nm. The titration curves were plotted by fluorescence emission intensities@740 nm versus pH. UV-vis-NIR absorption of the solutions was recorded as a function of buffer pH. The titration curves were plotted by absorbance@715 nm versus buffer pH.

pH dependent photothermal effects of SA-pNIR

Sodium phosphate buffer (pH 4.5 or pH 7.5) was spiked with or without SA-pNIR to a final concentration of 0.1 mg ml^{-1}. The solutions were irradiated with NIR light for 10 min (660 nm, 0.5 W cm^{-2}) and the temperature of the solutions was recorded over irradiation time.

NIR mediated photothermal effects in SA-pNIR treated cells

U87-MG cells, HeLa cells and Raw 264.7 cells were cultured for 24 h in DMEM supplemented with or without SA-pNIR (100 µg ml^{-1}) or SA (100 µg ml^{-1}) and irradiated with or without NIR laser (660 nm, 0.5 W cm^{-2}) for 10 min and then re-cultured for 24 h. The cell number and cell viability were determined by MTT assay.

Staining of lysosomes with SA-pNIR

HeLa cells, U87-MG cells and Raw 264.7 cells were respectively seeded on 35 mm glass-bottom dishes (NEST) and incubated for 24 h in DMEM supplemented with 10% FBS. The cells were cultured for 1 h in DMEM spiked with SA-pNIR (100 µM). The cells were washed with PBS (1 ml) and further incubated with Lysotracker green (1 µM) for 20 min in DMEM. The resultant cells were placed in fresh DMEM and then analyzed by confocal fluorescence microscopy.

Time-dependent cellualr uptake of SA-pNIR: HeLa, U87-MG (B) and Raw 264.7 cells (C) were respectively cultured with SA-pNIR (100 µM) in DMEM for 1 h, 4 h, or 24 h, and then stained with DiI (10 µM) for 5 min. The cells were then added in fresh DMEM. A portion of the cells were examined by confocal fluorescence microscopy for intracellular NIR signal over incubation time.

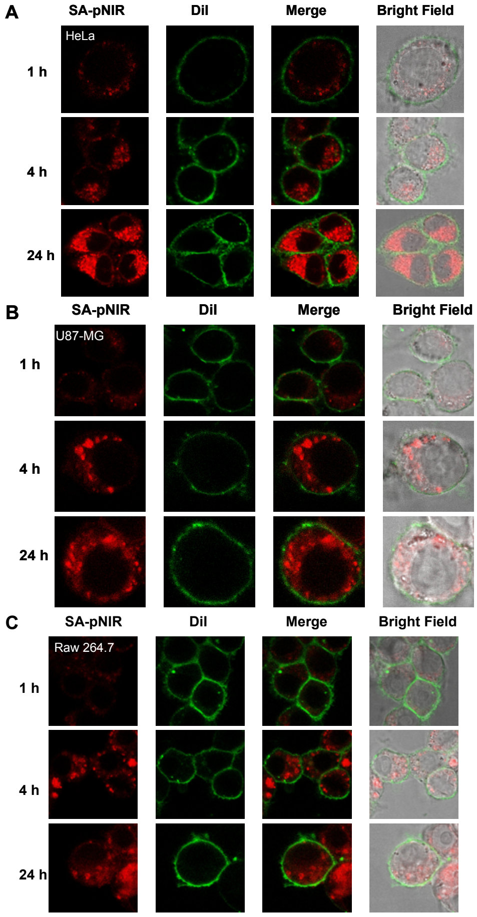

Fig. S1 Time-dependent uptake of SA-pNIR within cells. HeLa cells (A), U87-MG cells (B) and Raw 264.7 cells (C) were respectively cultured for 1-24 h with SA-pNIR (100 µM) in DMEM and then stained with DiI (10 µM) for 5 min. The cells were then cultured in fresh DMEM. A portion of the cells were examined by confocal fluorescence microscopy for intracellular NIR signal at indicated time points. The fluorescence of DiI was shown in green and that of SA-pNIR was shown in red. Bars, 10 µm.

Lysosomal activation of SA-pNIR

HeLa cells, U87-MG cells and Raw 264.7 cells were respectively seeded on 35 mm glass-bottom dishes (NEST) and incubated for 24 h in DMEM supplemented with 10% FBS. The cells were cultured in DMEM spiked with or without BFA (50 nM) for 30 min and then cultured in DMEM contining SA-pNIR (100 µM) for 1 h. The cells were further incubated in DMEM containing Lysotracker green (1 µM) for 20 min. A portion of the three cell lines pre-treated with BFA, SA-pNIR and Lysotracker green were incubated with sodium phosphate buffer (pH 4, 100 mM) for 10 min. The cells were probed by confocal fluorescence microscopy.

Metabolic incorporation of SA-pNIR into cellular proteins

HeLa, U87-MG and Raw 264.7 cells were respectively cultured with SA-pNIR (100 µM) in DMEM for 24 h and stained with DiI (10 µM) for 10 min. The cells were treated with acetone for 5 min at 4 ℃ and 10 min at 37 ℃ in PBS, incubated with fresh DMEM (pH 7.4) or sodium phosphate buffer (pH 4.0, 100 mM) for 10 min, and then imaged by fluorescence confocal microscopy.

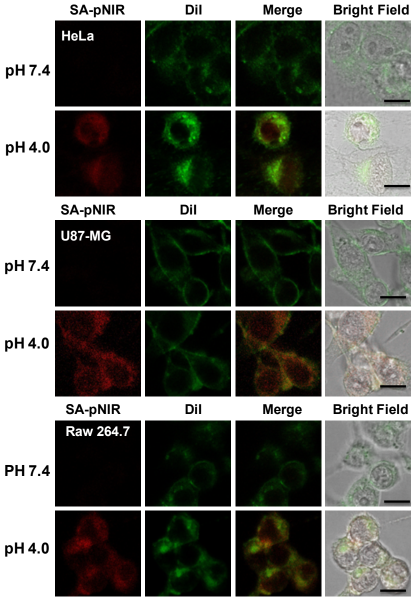

Fig. S2 Metabolic incorporation of SA-pNIR into cellular proteins. HeLa cells (A), U87-MG cells (B) and Raw 264.7 cells (C) were respectively cultured with SA-pNIR (100 µM) for 24 h and stained with DiI (10 µM) for 10 min. The cells were treated with acetone for 5 min at 4 ℃ and 10 min at 37 ℃ in PBS, incubated with fresh DMEM (pH 7.4) or sodium phosphate buffer (pH 4.0, 100 mM) for 10 min, and then imaged by fluorescence confocal microscopy. The cell membrance stained with DiI was shown in green and the intracellular SA-pNIR was shown in red. Bars, 10 µm.

Imaging of subcutaneous tumors in mice with SA-pNIR

Nude mice were xenografted in the flank by subcutaneous injections of H22 cells (1x10^{6}). At 5-8 days after the transplantation, SA-pNIR (40 mg kg^{-1}) were respectively injected intravenously via the tail vein into tumor-bearing mice. At 10 min to 144 h following injection, the mice were analyzed for whole body NIR fluorescence.

In vivo distribution of SA-pNIR in tumor-bearing mice

A cohort of ICR mice with subcutaneous injections were injected intravenously via the tail vein with SA-pNIR (7.5 mg kg^{-1} or 35 mg kg^{-1}) or GLu-pNIR (7.5 mg kg^{-1} or 35 mg kg^{-1}). At 48 h or 114 h following injection, the mice were anesthetized and then analyzed for whole body NIR signals. The anesthetized mice were then sacrificed and the tumors and selected organs were excised, washed with PBS and subjected to *ex vivo* analysis for the NIR fluorescence.

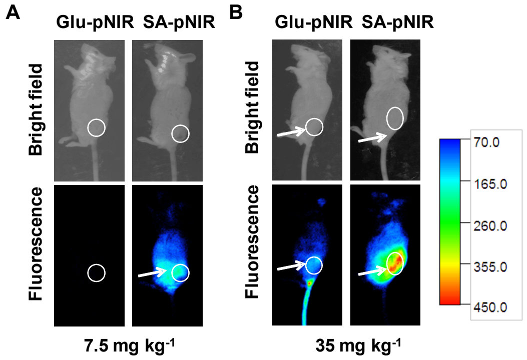

Fig. S3 Whole body fluorescence imaging of tumor-bearing ICR mice with SA-pNIR or Glu-pNIR. Tumor-bearing ICR mice were intravenously injected with SA-pNIR or Glu-pNIR with the doses of 7.5 mg kg^{-1} (A) or 35 mg kg^{-1} (B) and then imaged 114 h postinjection.

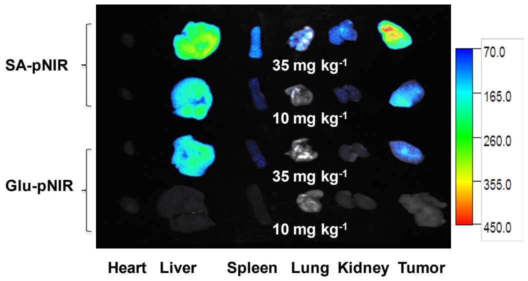

Fig. S4 Biodistribution of SA-pNIR or Glu-pNIR in tumor-bearing mice. Tumor-bearing ICR mice were intravenously injected with SA-pNIR (7.5 mg kg^{-1} or 35 mg kg^{-1}) or Glu-pNIR (7.5 mg kg^{-1} or 35 mg kg^{-1}) *via* tail vein. At 114 h postinjection, the tumor and representative organs were dissected and then imaged for *ex vivo* fluorescence emission.

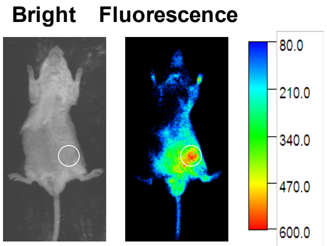

Fig. S5 Whole body detection of subcutaneous tumors with over-dosed SA-pNIR. ICR mice was intravenously injected with SA-pNIR (150 mg kg^{-1}) *via* tail vein and then imaged for *in vivo* NIR fluorescence emission 10 days postinjection.

Cytotoxicity of SA-pNIR

For cell toxicity: HeLa cells were respectively cultured for 24 h in DMEM medium containing various levels of SA-pNIR (0-100 µg ml^{-1}). The cell number and cell viability were determined by trypan blue exclusion assay.

For systemic toxicity: ICR mice bearing subcutaneous tumor were intravenously injected with SA-pNIR (150 mg kg^{-1}) and was regularly monitored for abnormality following injection. The mice were anesthetized and sacrificed at the 10th day postinjection. The tumor and representative organs were dissected and then imaged for *ex vivo* fluorescence emission.

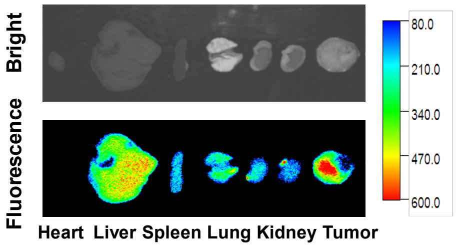

Fig. S6 Biodistribution of over-dosed in tumor-bearing mice. ICR mice bearing subcutaneous tumor were intravenously injected with SA-pNIR (150 mg kg^{-1}) and then sacrificed 10 days postinjection. The tumor and representative organs were dissected and then imaged for *ex vivo* fluorescence emission.
